# Metabolite Profiling in Green Microalgae with Varying Degrees of Desiccation Tolerance

**DOI:** 10.3390/microorganisms10050946

**Published:** 2022-04-30

**Authors:** Siegfried Aigner, Erwann Arc, Michael Schletter, Ulf Karsten, Andreas Holzinger, Ilse Kranner

**Affiliations:** 1 Department of Botany, University of Innsbruck, Sternwartestraße 15, A-6020 Innsbruck, Austria; si.aigner@tsn.at (S.A.); erwann.arc@uibk.ac.at (E.A.); michael.schletter@web.de (M.S.); andreas.holzinger@uibk.ac.at (A.H.); 2 Institute of Biological Sciences, University of Rostock, Albert-Einstein-Straße 3, D-18057 Rostock, Germany; ulf.karsten@uni-rostock.de

**Keywords:** *Chlorella*, *Diplosphaera*, *Edaphochlorella*, green algae, microalgae, metabolite, metabolomics

## Abstract

Trebouxiophyceae are microalgae occupying even extreme environments such as polar regions or deserts, terrestrial or aquatic, and can occur free-living or as lichen photobionts. Yet, it is poorly understood how environmental factors shape their metabolism. Here, we report on responses to light and temperature, and metabolic adjustments to desiccation in *Diplosphaera epiphytica*, isolated from a lichen, and *Edaphochlorella mirabilis*, isolated from Tundra soil, assessed *via* growth and photosynthetic performance parameters. Metabolite profiling was conducted by GC–MS. A meta-analysis together with data from a terrestrial and an aquatic *Chlorella vulgaris* strain reflected elements of phylogenetic relationship, lifestyle, and relative desiccation tolerance of the four algal strains. For example, compatible solutes associated with desiccation tolerance were up-accumulated in *D. epiphytica*, but also sugars and sugar alcohols typically produced by lichen photobionts. The aquatic *C. vulgaris*, the most desiccation-sensitive strain, showed the greatest variation in metabolite accumulation after desiccation and rehydration, whereas the most desiccation-tolerant strain, *D. epiphytica*, showed the least, suggesting that it has a more efficient constitutive protection from desiccation and/or that desiccation disturbed the metabolic steady-state less than in the other three strains. The authors hope that this study will stimulate more research into desiccation tolerance mechanisms in these under-investigated microorganisms.

## 1. Introduction

Chlorophyte microalgae are receiving increasing attention due to their contribution to carbon and nitrogen cycling in soil crusts [1,2] and as resources for valuable compounds [3]. However, it is still poorly understood how they adjust their metabolism in response to environmental stress factors that impact on their growth and ecophysiological performance. In a previous study [4], ecophysiological traits and metabolite composition of two *Chlorella vulgaris* strains, one aquatic and one terrestrial, were investigated. In this study, the ecophysiological performance under different light and temperature conditions, and their ability to recover from desiccation of two phylogenetically well-characterized Trebouxiophycean green algae in the Prasiola-clade, *Edaphochlorella mirabilis* (SAG 38.88) isolated from Tundra soil, and *Diplopshaera epiphytica* (SAG 11.88) isolated from the lichen *Pseudocyphellaria* *carpoloma* [5,6,7,8], were investigated. Trebouxiophyceae are widespread globally, and occur in many aquatic and terrestrial ecosystems [9,10,11] with different lifestyles [2,12] either free-living or as lichen photobionts [13,14,15]. Ecophysiological traits of *D. epiphtica* and *D. chodatii* were recently reported [16,17,18]. In *D. epiphtica*, sorbitol and sucrose contents were used as chemotaxonomic markers [16], and the potential of *Diplopshaera* spp. for biotechnological applications was investigated [19]. Furthermore, molecular markers of the lichen photobiont, *D. chodatii*, were used to study dispersal modes of the semi-aquatic lichen, *Dermatocarpon luridum* [20]. Comparably little is known for *Edaphochlorella* spp., e.g., ecophysiological studies were conducted with arctic strains [21,22]. In summary, some reports on the ecophysiological characteristics of *Diplopshpaera* and *Edaphochlorella* species exist, but hardly any information is available on changes in metabolism in response to environmental stress factors.

Compared to aquatic species, aero-terrestrial algae are exposed to greater variations in temperature, higher irradiation, and a desiccating atmosphere. Mechanisms that protect aero-terrestrial chlorophytes from these environmental factors include photoprotectants and osmoprotectants [23,24], and/or self-shading through the formation of cell aggregates (e.g., *Apatococcus* spp.) or cell colonies (e.g., *Coccomyxa* spp.) [25]. In addition, the excretion of extracellular mucilage or the formation of multi-layered filaments (e.g., *Klebsormidium* spp.) may contribute to a protective matrix that retains water [26], whereas other microorganisms are capable of surviving desiccation.

Desiccation tolerance can be defined as the ability to revive from the air-dried state or, *sensu stricto*, as the capability to survive drying at relative humidity below 65%, corresponding to a drop in absolute water contents to or below 0.1 g H_2_O g^–1^ dry mass and a water potential of ≤−100 MPa [27,28,29]). In two microalgae isolated from lichens, *Trebouxia* sp. and *Coccomyxa solorina-saccatae*, the significance of polyols (sugar alcohols containing multiple hydroxyl groups) for survival of desiccation was highlighted [30]. However, sugar alcohols also serve as important substrates for growth and respiration shunted between the algal (or cyanobacterial) and fungal partners [31,32], and it should be noted that desiccation tolerance is based on a plethora of protection mechanisms. For review of the constitutive and inducible protection mechanisms associated with biochemical and morphological modifications supporting survival in the desiccated state in resurrection plants, mosses, and lichens, see [33,34,35]. Among the various desiccation-tolerant taxa, green algae are under-investigated, and with the exception of lichen photobionts, it is not even clear which chlorophytes are desiccation-tolerant (for review, see [36,37]).

Moreover, it remains an enigma why some phylogenetically closely related green algae vary strongly in their desiccation tolerance [36]. For example, in *Tetradesmus* spp., desiccation tolerance appears to be a common trait in temperate and desert soil crusts, but is missing in closely related aquatic species [38]. Differences in relative desiccation tolerance were also observed in closely related species, or even within the same species, in the Chlorellaceae and Scenedesmaceae [4,25,39]. *Diploshaera chodatii* was described as desiccation-tolerant, capable of recovering from desiccation at 10% relative humidity [18].

In the present paper, we made use of previously published data on the ecophysiology and the effects of desiccation and rehydration on differential metabolite accumulation in the above-mentioned *C. vulgaris* strains, one of which was desiccation-sensitive and the other capable of surviving desiccation at 84% relative humidity, in comparison with *D. epiphytica* and *E. mirabilis*. The central research aims were, first, to study the ecophysiological performance and primary metabolism of *D. epiphytica* and *E. mirabilis* in response to desiccation and rehydration; and second, to conduct a meta-analysis of the metabolite data gained together with those from both *C. vulgaris* strains [4], to obtain deeper insights into the metabolite composition in microalgae with different degrees of desiccation tolerance.

## 2. Materials and Methods

### 2.1. Algal Strains, Culture Conditions, and Microscopy

The algal strains *Edaphochlorella mirabilis* (SAG 38.88), Trebouxiophyceae *incertae sedis*, and *Diplosphaera epiphytica*, Stichococcaceae (formerly *Chlorella sphaerica*; SAG 11.88), both in the *Prasiola* clade [6,8] were obtained from the Culture Collection of Algae at Göttingen University, Germany (SAG). Cell size was investigated by a Zeiss Axiovert 200 M microscope, equipped with a 63 × (1.4 numerical aperture) objective lens, showing that young cells were ellipsoidal and mature cells were spherical in both species, with a mean cell size of 5.01 ± 0.53 and 7.44 ± 1.19 μm (*n* = 20 cells) in *D. epiphytica* and *E. mirabilis*, respectively.

Algae were cultivated in liquid Bolds Basal Medium plus vitamins (BBM + V) [40] under a 16 h light (20–25 µmol photons m^−2^ s^−1^ at 20 °C)/dark (8 h at 15 °C) cycle. Log-phase cultures were used in all experiments.

### 2.2. Light and Temperature Treatments, and Temperature-Dependent Oxygen Production/Consumption

To link the ecophysiological performance of *D. epiphytica* and *E. mirabilis* under different experimental light and temperature conditions to previously published data [4], the effects of light and temperature on growth were assessed using the same methods. Briefly, using a 16 h light/8 h dark cycle, growth rates were monitored in response to different photon fluence densities (PFDs) at 15 °C, and to different temperatures at 20–25 μmol photons m^−2^ s^−1^, using the increase in chlorophyll *a* fluorescence over time as an indicator of biomass accumulation as previously described [4].

Photosynthetic oxygen production rates at temperatures from 5 °C to 50 °C were measured with a Fibox 3 oxygen optode (Presens, Regensburg, Germany) after [41], as previously described [4], whereby oxygen production (gross photosynthesis) and consumption (respiration) were normalized to the concentration of total chlorophyll *a*, analysed, and calculated as previously described [42].

### 2.3. Desiccation and Rehydration Treatments

Algae were subjected to a desiccation and rehydration treatment exactly as previously described [4]. Briefly, 100 µL of cell suspensions were transferred onto 8 mm Whatman GF/F glass fibre filters (Whatman, Dassel, Germany) and grown for 5 additional days on solidified 1.25% agar containing BBM + V. Then, filters with 0.75 ± 0.05 mg DW^−1^, determined after freeze-drying (Lyovac GT2, Leybold, Köln, Germany) of algae, were wetted with 20 µL of BBM + V before placing them in a desiccation chamber above 100 mL of saturated KCl solution, resulting in a final relative air humidity (RH) of 84% inside the chamber at ambient temperature (22 ± 1 °C) and 20 µmol photons m^−2^ s^−1^. Cultures were exposed to 84% RH for 180 min, and then rehydrated by pipetting 20 µL of liquid BBM + V medium onto each filter and replacing the KCl solution with *A. dest.*, resulting in 95% RH within the chamber.

To noninvasively monitor the algae’s response to desiccation and to assess their ability to recover from desiccation, the effective quantum yield Y(II) was measured using a pulse-amplitude-modulated (PAM) fluorimeter (PAM 2500, Heinz Walz GmbH, Effeltrich, Germany). Data were recorded at intervals during desiccation until no effective quantum yield Y(II) was detected, and during approximately 17 h of rehydration (1042 and 1044 min for *D. epiphytica* and *E. mirabilis*, respectively; *n* = 5 filters per interval).

In addition, electron transport rates (ETRs) were measured before desiccation (controls) and at the end of the rehydration experiment (*n* = 4 filters). For this, algae were 30 min dark-adapted on filters placed on agar plates and exposed to 16 PFDs from 0 to 1660 μmol photons m^−2^ s^−1^ (for 30 s each) as previously described [43].

### 2.4. GC–MS-Based Metabolite Profiling

Algal cultures on filters prior to desiccation (controls) and at the end of the rehydration experiment (*n* = 3 filters each) were frozen in liquid nitrogen and freeze-dried. Freeze-dried material was ground with glass beads using a laboratory mill (Tissuelyser II, Qiagen, Venlo, the Netherlands) at 30 Hz for 3 min before metabolite extraction.

Metabolite profiling was carried out using the slightly modified method of [44] as previously described [4], and samples were processed together with the *C. vulgaris* samples described in [4]. Briefly, aliquots of freeze-dried and finely ground material and quality controls, including commercially available standards and blanks, were extracted at −20 °C in water:acetonitrile:isopropanol (2:3:3) containing ^13^C_6_-sorbitol and ^13^C_5_, ^15^N-valine as internal standards. After a centrifugation step, an aliquot of the supernatant was collected and dried in a vacuum centrifuge, and metabolites were derivatized with methoxyamine in pyridine solution and N-methyl-N-trimethylsilyl-trifluoroacetamide (MSTFA). Metabolites were separated on a 30 m Rxi-5Sil MS column with a 10 m Integra-Guard pre-column (Restek) using a Trace 1300 gas chromatograph in splitless mode and detected by a TSQ8000 triple quadrupole mass spectrometer (Thermo Scientific, Waltham, MA, USA). A mix of alkanes was injected in the middle of the queue for external retention index calibration. Compound spectra were extracted from the raw data files using the “Automated Mass-spectral Deconvolution and Identification System” (AMDIS) and compared against a custom-built mass spectral library and commercial or publicly available databases, including the NIST, Golm, and Fiehn databases [45,46]. Peak areas for compound-specific trace ions were determined using the Xcalibur software (Thermo Scientific) for relative quantification of identified and unknown compounds in the biological samples.

In addition, in untreated algae, proline contents were quantified spectrophotometrically [47], and sorbitol [23] and prasiolin by HPLC, the latter using prasiolin isolated from the green alga *Prasiola calophylla* for quantification [48].

### 2.5. Statistical Analysis

Statistical evaluation of the data was performed with R (R Core Team, 2022) using the ellipse, lsmeans [49], and ggplot2 packages [50]. Physiological data were tested for significance by two-way ANOVA, followed by Tukey’s multiple comparison test. Subgroups with significantly different means were identified at *p* < 0.05. Metabolites were reported as differentially accumulated between the strains when the false discovery rate (FDR [51])-corrected Welch ANOVA *p* value was below 0.01 with a log_2_ ratio higher than 1. Whenever the ANOVA assumption of normal distribution was not fulfilled, even after Box–Cox transformations (e.g., log or square root transformation), nonparametric tests (pairwise Mann–Whitney U tests or Kruskal–Wallis tests) were applied instead. For each strain, metabolites were reported as differentially accumulated after desiccation and rehydration when the FDR-corrected Welch T-test *p* value was below 0.05.

## 3. Results

### 3.1. Effects of Light and Temperature on Growth Rates, and Temperature-Dependent Oxygen Production

**Figure 1:**
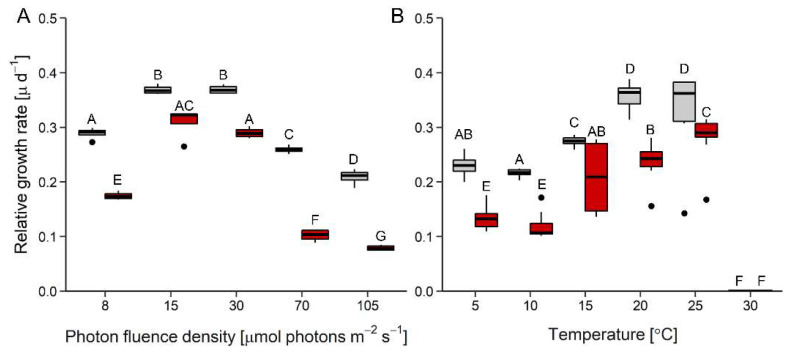
Effects of photon fluence density and temperature on growth. Panels (**A**) and (**B**) show relative growth rates as a function of increasing photon fluence density (*n* = 4) and temperature (*n* = 8), respectively, in *Diplosphaera epiphytica* (red) and *Edaphochlorella mirabilis* (grey). Boxplots show minima and maxima (whiskers), 25th percentiles, medians, and 75th percentiles, and dots are outliers. In each panel, different capital letters indicate significant differences, calculated by pairwise Mann–Whitney U-tests after FDR correction (*p* < 0.05).

Growth rates were affected by both photon fluence density (PFD) (Figure 1A) and temperature (Figure 1B), whereby *D. epiphytica* showed consistently lower growth rates than *E. mirabilis*. In response to increasing irradiation, both species showed maximum growth rates between 15 and 30 μmol photons m^−2^ s^−1^, and growth rates declined at higher PFDs. At 105 μmol photons m^−2^ s^−1^, growth rates were reduced by two-thirds compared to maximum values in *D. epiphytica*, but only by about 40% of maximum values in *E. mirabilis* (Figure 1A). In response to increasing temperature, no differences in growth rates were found at 5 and 10 °C, and then growth rates significantly increased with temperature up to a maximum at 20 °C, remaining stable at 25 °C, and no growth was recorded in both species at 30 °C (Figure 1B).

**Figure 2:**
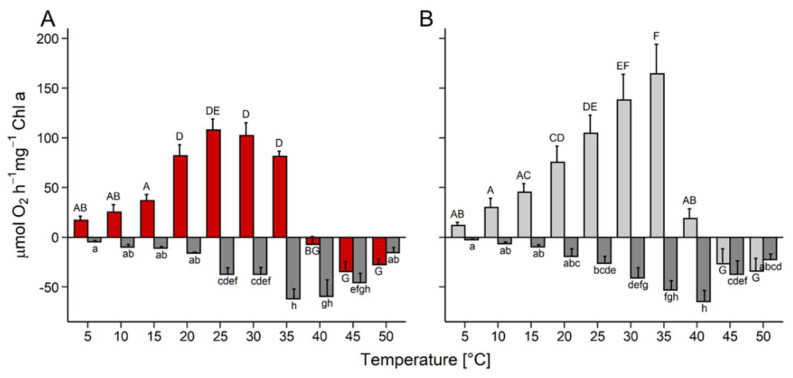
Effects of temperature on photosynthesis and respiration. Gross photosynthetic oxygen production and respiratory oxygen consumption were measured at 200 µmol photons m^−2^ s^−1^ as a function of increasing temperature in (**A**) *Diplosphaera epiphytica* and (**B**) *Edaphochlorella mirabilis*. Red and light grey bars show gross photosynthetic oxygen production in (**A**) and (**B**), respectively, and dark grey bars show respiratory oxygen consumption in both panels; data are means ± SD (*n* = 4). Different small (respiratory oxygen consumption) and capital (gross photosynthesis) letters indicate significant differences between temperature treatments and species, and were calculated by two-way ANOVA followed by Tukey’s *post hoc* test (*p* < 0.05).

Oxygen production of *D. epiphytica* did not change from 5 to 15 °C, and then increased significantly, with a plateau between 20 °C and 35 °C, before ceasing at 40 °C (Figure 2A). In *E. mirabilis*, gross oxygen production almost linearly increased between 5 and 35 °C, was reduced by approximately 90% at 40 °C, and ceased at 45 °C (Figure 2B). In both strains, respiratory oxygen consumption was very low at 5 °C, then slowly increased to maximum values at 35 °C and 40 °C in *D. epiphytica* and *E. mirabilis*, respectively, then declined at higher temperatures (Figure 2).

### 3.2. Effects of Desiccation and Rehydration Treatments

**Figure 3:**
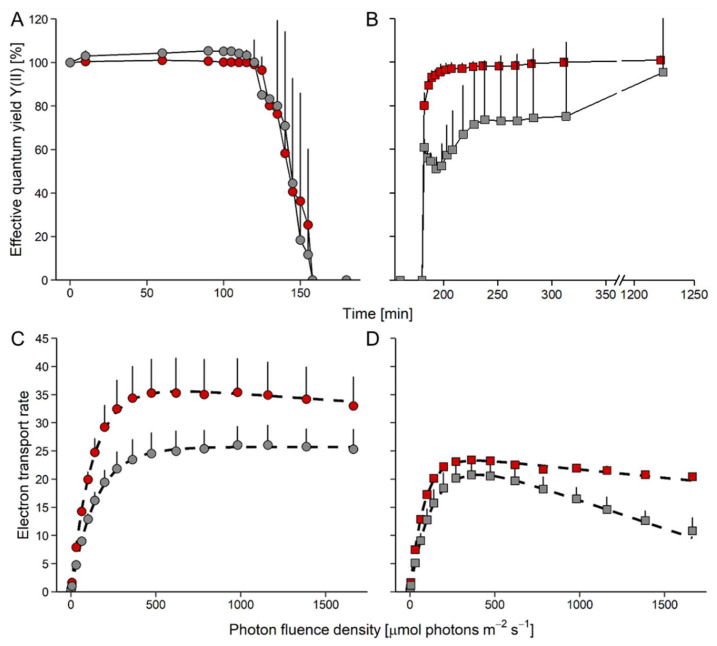
Effects of desiccation and rehydration on photosynthesis in *Edaphochlorella mirabilis* and *Diplosphaera epiphytica*. Red symbols denote *D. epiphytica* and grey symbols show *E. mirabilis*. (**A**) Effective quantum yield during desiccation under 84% relative humidity and (**B**) during the subsequent rehydration under 95% relative humidity; data are means ± SD (*n* = 5) expressed as a percentage of the initial values at time 0. (**C**) Electron transport rates of algae before desiccation and (**D**) after the end of rehydration; data are means ± SD (*n* = 4). Two-way ANOVA (*p* < 0.05) showed that the two species in panels (**B**–**D**) differed significantly.

Upon exposure to 84% RH, the YII did not change for 120 min, and then decreased to zero within the next 30 min in both species (Figure 3A). Thereafter, the cultures were kept at 84% RH for another 30 min, and upon subsequent rehydration (Figure 3B), YII rapidly returned to the levels of nondesiccated cultures in *D. epiphytica*, reaching 95% of the initial YII within 20 min. By contrast, *E. mirabilis* recovered only to about 50% of the YII values of nondehydrated cells after 20 min, and only after rehydration for 17 h, 95% of the initial value was re-gained (Figure 3B).

In nondesiccated controls, ETRs increased in *D. epiphytica*, with some slight photoinhibition at PFDs above 500 μmol photons m^−2^ s^−1^ (Figure 3C; characteristic parameters of the ETR curve, ETR_max_, I_k_, and α, are given in Table S1). In *E. mirabilis*, ETR values increased up to 1600 μmol photons m^–2^ s^–1^. Nondesiccated *D. epiphytica* cultures had higher ETR_max_ values when compared with *E. mirabilis*; however, the I_k_ of *D. epiphytica* was lower than that of *E. mirabilis*. At the end of the rehydration experiment (Figure 3D), ETR_max_ values of cultures of both species were significantly lower compared to nondesiccated cultures (Figure 3C). Both species showed photoinhibition, which was more pronounced in *E. mirabilis* than in *D. epiphytica*. After the desiccation-rehydration treatment, ETR_max_ and I_k_ values were significantly lower in both species.

### 3.3. Meta-Analysis of Metabolites: Constitutive Differences between the Four Algal Strains

**Figure 4:**
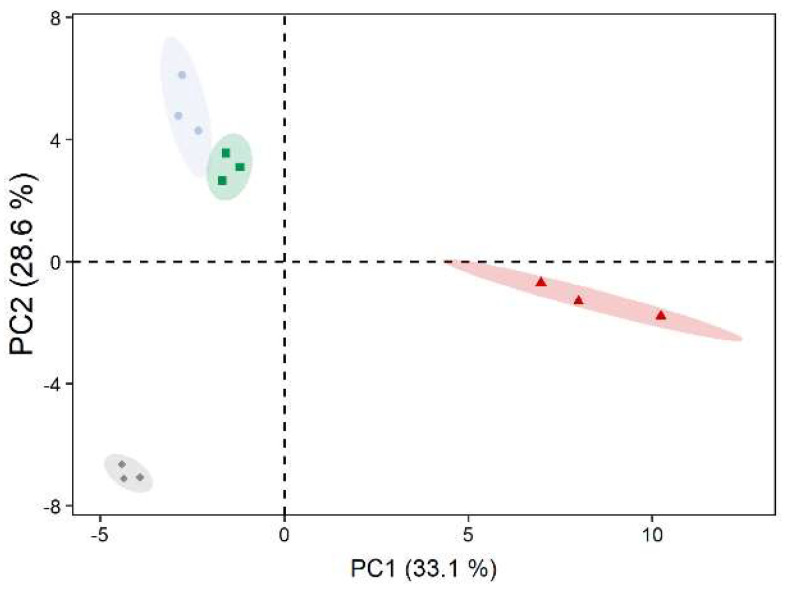
Principal component analysis of metabolite profiles of untreated algae. *Diplosphaera epiphytica* (red triangles), *Edaphochlorella mirabilis* (grey diamonds), and two *Chlorella vulgaris* strains (aquatic *C. vulgaris*: blue circles, terrestrial *C. vulgaris*: green squares) were compared; *n* = 3 biological replicates. Relative abundances for the 75 identified metabolites were used after mean centring.

A total of 75 metabolites were identified using GC–MS-based metabolite profiling. Principal component analysis showed a clear separation between the metabolite profile of *D. epiphytica* and those of the other three strains along principal component 1 (PC1), accounting for 33% of the total variance. The two *C. vulgaris* strains were further separated from *E. mirabilis* separated along PC 2, accounting for 29% of the variance (Figure 4).

**Figure 5:**
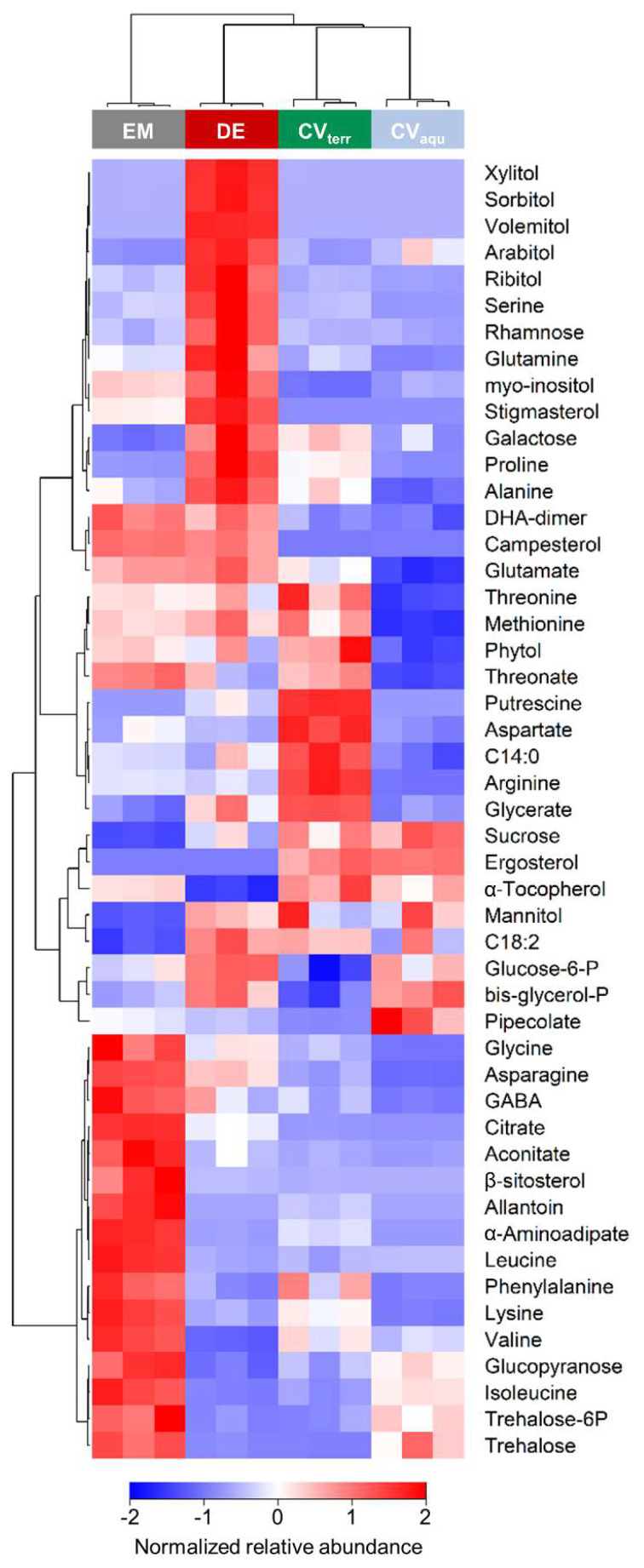
Differences in metabolite accumulation between the four algal strains. The heatmap shows a comparison of untreated algae; colours from dark to light blue, via white, to light to dark red denote low to high relative abundances. Colour code on top: grey, blue, red, and green denote *Edaphochlorella mirabilis* (EM), the aquatic strain of *Chlorella vulgaris* (CV_aqu_), *Diplosphaera epiphytica* (DE), and the terrestrial strain of *Chlorella vulgaris* (CV_terr_), respectively, according to one-way ANOVA *p*-value < 0.01 after FDR correction, max log_2_ ratio > 1.

Out of the four strains, *D. epiphytica* had the highest contents of various sugars and sugar alcohols, including galactose and rhamnose, xylitol, sorbitol, volemitol, arabitol, myo-inositol, and ribitol, and several amino acids such as serine, glutamine, proline, and alanine (Figure 5 and Table S3). In contrast, *E. mirabilis* showed the highest contents of several amino acids, as well as citrate, β-sitosterol, allantoin, trehalose, and trehalose-6-P, but had the lowest sucrose, mannitol, and linoleic acid contents. In the terrestrial *C. vulgaris* strain, putrescine, aspartate, arginine, and glycerate contents were higher compared to the other three strains. In addition, metabolites that were present at higher levels in the two *C. vulgaris* strains, compared to *D. epiphytica* and *E. mirabilis*, included sucrose and ergosterol, whereas glycine, asparagine, DHA, and campesterol were lower. Overall, the aquatic *C. vulgaris* strain had the lowest amino acid contents, but higher pipecolic acid contents. Two free fatty acids, myristic acid (C 14:0) and linoleic acid (C 18:2), were also differentially accumulated.

Furthermore, a spectrophotometric assay for proline confirmed that the concentration of this osmolyte was at least two-fold higher in *D. epiphytica* than in *E. mirabilis* (Table S2). Sorbitol was detected by HPLC analysis in *D. epiphytica*, but not in *E. mirabilis*. Prasiolin, a mycosporine-like amino acid, was detected in both algae, where its concentration was nearly five times higher in *D. epiphytica* than in *E. mirabilis* (Table S2).

### 3.4. Meta-Analysis of Metabolites: Differences between the Four Algal Species in Response to Desiccation and Rehydration

**Figure 6:**
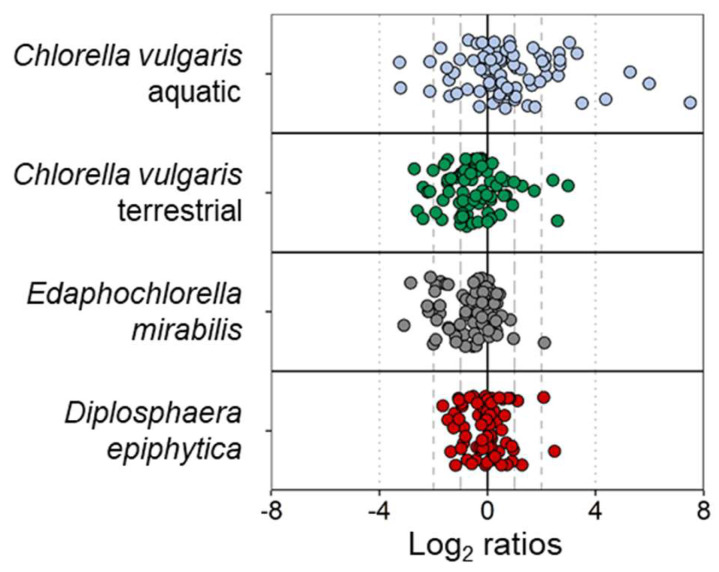
Changes in metabolite profiles before and after desiccation and rehydration in each of the four algal strains. Out of a total of 75 metabolites, 13, 22, 25, and 38 metabolites showed an absolute log_2_ ratio above 1 in *Diplosphaera epiphytica* (red), *Edaphochlorella mirabilis* (grey), the terrestrial *Chlorella vulgaris* (green), and the aquatic *Chlorella vulgaris* strain (blue), respectively.

Out of the four strains, *D. epiphytica* showed the smallest variation in its metabolite profile following the desiccation-rehydration treatment, and only 13 metabolites showed a fold change over a log ratio of 1 (Figure 6, Table S3). By contrast, the aquatic *C. vulgaris* strain exhibited the largest variation in its metabolite profile, whereby 38 metabolites showed a fold change over a log ratio of 1.

**Figure 7:**
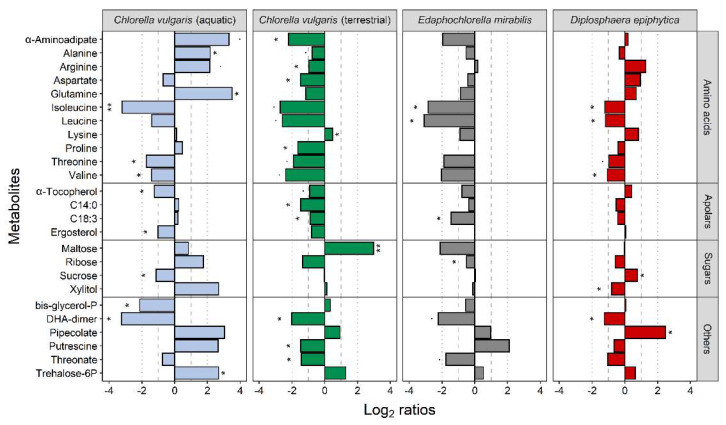
Effects of desiccation and rehydration on the metabolite profiles of the four algal strains. Blue, green, grey, and red colours indicate the aquatic and the terrestrial *Chlorella vulgaris* strains, *Edaphochlorella mirabilis* and *Diplosphaera epiphytica*, respectively. Previously published datasets for the two *C. vulgaris* strains [4] were used for meta-analysis. The effects of the desiccation-rehydration treatment on metabolite accumulation are shown as log_2_ ratios (metabolite contents after the desiccation-rehydration treatment divided by the metabolite contents in nondesiccated controls). A positive log_2_ ratio indicates that a given metabolite was up-accumulated after the treatment and a negative log_2_ ratio indicates that it was down-accumulated. Differences are shown for all metabolites differentially accumulated after the treatment as compared to the control (FDR-corrected Welch *t*-test at *p* < 0.05; log_2_ ratios > 1) in at least one of the strains, with one and two asterisks denoting adjusted *p*-values below 0.05 and 0.01, respectively, for the given comparison.

Twenty-five metabolites were significantly differentially accumulated in at least one of the four strains (Figure 7). The terrestrial *C. vulgaris* strain and *E. mirabilis* revealed very similar patterns of differential accumulation, and most of these 25 metabolites were down-accumulated after the desiccation-rehydration treatment. Exceptions from this common pattern between the terrestrial *C. vulgaris* strain and *E. mirabilis* were putrescine, maltose, and lysine. Putrescine was up-accumulated in *E. mirabilis* and down-accumulated in the terrestrial *C. vulgaris* strain after treatment, and *vice versa* in maltose and lysine. The aquatic *C. vulgaris* strain showed the highest number of up-accumulated metabolites, including α-aminoadipate, alanine, arginine, glutamine, putrescine, trehalose-6-phosphate, and xylitol. Two free fatty acids were also up-accumulated in *C. vulgaris*, compared to the other strains. Of the metabolites differentially accumulated in *D. epiphytica* and the aquatic *C. vulgaris* was sucrose, which was unaffected in *E. mirabilis* and the terrestrial *C. vulgaris* strain (Figure 7).

## 4. Discussion

This study reports on the potential to recover from a desiccation-rehydration treatment of four algal strains with varying degrees of desiccation tolerance, conducting a meta-analysis of the *D. epiphytica* and *E. mirabilis* data compared to previously published metabolite data from a terrestrial and an aquatic strain of *C. vulgaris* [4]. For this, we first characterized ecophysiological traits of the first two species to enable comparison with the *C. vulgaris* data, and then investigated constitutive differences in the metabolite profiles of the four strains and in response to the desiccation-rehydration treatment.

Assessing the ecophysiological performance of *D. epiphytica* and *E. mirabilis* revealed differences between the two species in light and temperature requirements for growth, and in their tolerance of desiccation. *Edaphochlorella mirabilis* grew better than *D. epiphytica* in response to increasing irradiation and temperature (Figure 1), in agreement with the capability to grow under a relatively broad temperature range reported for *E. mirabilis* extracted from polar soil [21]. Furthermore, gross oxygen production increased with temperature up to 35 °C in *E. mirabilis* and to 25 °C in *D. epiphytica* (Figure 2). Tolerance of low rather than high temperatures has been reported for lichen photobionts [52,53], which are known to be adapted to low PFDs [54]. A previous study [55] also reported that two lichen photobionts were more sensitive to light than four strains of free-living algae. However, it was not the aim of this study to conclude on the prerequisites required for a free-living alga to become a lichen photobiont, which would entail a comprehensive study of many strains of free-living and lichenized algae. In response to a desiccation and rehydration treatment, ETR_max_ was lower compared to nondesiccated controls and *E. mirabilis* showed more photoinhibition than *D.* *epiphytica* (Figure 3C,D). However, *D. epiphytica* appeared to be more tolerant of desiccation than *E. mirabilis*, assessed by the faster recovery of YII in *D. epiphytica* compared to *E. mirabilis* (Figure 3A,B). The higher relative desiccation tolerance of *D. epiphytica* is consistent with a report on *D. chodatii*, which was able to survive desiccation at 10% relative humidity [18].

In summary, *D. epiphytica* was more sensitive to light and temperature, but more tolerant of desiccation than *E. mirabilis*. Furthermore, the four strains showed different kinetics of YII recovery, especially in the beginning of rehydration. The aquatic *C. vulgaris* strain recovered only 30% of YII values of nondesiccated controls within the first 20 min of rehydration, whereas the terrestrial strain recovered about 80% [4]. *Diplosphaera epiphytica* recovered even faster, reaching 95% of YII values of nondesiccated controls within the first 20 min, whereas *E. mirabilis* recovered only about 50% (Figure 3A,B). Overall, the aquatic *C. vulgaris* strain appears to be the least desiccation-tolerant of the four strains and the lichen photobiont *D. epiphytica* the most desiccation-tolerant one.

### 4.1. Constitutive Differences in the Metabolite Profiles of the Four Algal Strains

PCA clearly separated *D. epiphytica* from the other three strains based on their metabolite profiles (Figure 4). Compared to the other three strains, *D. epiphytica* had higher contents of several sugars and sugar alcohols, such as sorbitol, ribitol, and xylitol, and other compatible solutes such as proline (Figure 5). Proline, together with sorbitol, is among the key compatible solutes associated with protection from damage induced by various stress factors interfering with osmoregulation, such as dehydration [56,57]. The higher sorbitol and proline contents in *D. epiphytica* were confirmed using an HPLC assay and a spectrophotometric assay, respectively (Table S2), in agreement with previous work on the same strain (SAG 11.88) and *D. chodatii*, showing that sorbitol is present at high concentrations [16,17,18]. Several sugars and sugar alcohols, including sorbitol, have been suggested to play a role in desiccation tolerance [30,32], but they also play a role in the lichen symbiosis [31,32,58]. *Diplosphaera* spp. are widespread lichen photobionts, for example, in the Verrucariaceae [13]. Sugars and sugar alcohols produced by lichen photobionts represent the main carbon source for their fungal partners (i.e., their mycobionts), which can convert them into other sugars and sugar alcohols [58,59,60,61]. For example, ribitol, erythritol, sorbitol, and glucose are produced by lichen photobionts and transported to their corresponding mycobionts in a broad range of lichen species [62,63], and glucose, sucrose, and rhamnose were also found to be extracellularly released by isolated lichen photobionts, suggesting potential physiological roles [58,64]. Compared to the other three strains, several amino acids such as serine, glutamine, proline, and alanine were also up-accumulated in *D. epiphytica*. Recently, it has been suggested that sugars, typically found to increase to high concentrations during dehydration of vegetative desiccation-tolerant tissues, together with organic acids and amino acids, could contribute to the formation of “natural deep eutectic solvents” (NADES), which are “functional liquid media” capable of dissolving chemicals of low water solubility [65]. This could support survival in the desiccated state, and it would be interesting to test this assumption in future studies. In summary, the higher contents in compatible solutes, such as sorbitol and proline, in *D. epiphytica* support the finding that this alga is the most desiccation-tolerant of the four strains investigated, and produces sugars and sugar alcohols that are favoured carbon sources for lichenized fungi [66,67], making it a good candidate photobiont.

*Edaphochlorella mirabilis* had low sucrose contents compared to the other three strains (Figure 5), which could point at a lower photosynthetic carbon assimilation, consistent with its lower ETR values compared to *D. epiphytica* (Figure 3) and the two *C. vulgaris* strains [4]. Furthermore, trehalose and trehalose-6-P, an intermediate of trehalose biosynthesis, were up-accumulated in *E. mirabilis* and, to a lesser extent, also in the aquatic *C. vulgaris* strain. In plants, trehalose-6-phosphate is thought to be a signal of sucrose status and a regulatory molecule associated with sugar influx and metabolism [68]. Trehalose has also been suggested to confer desiccation tolerance, although this role has been disputed [69]. A study with yeast mutants that do not produce trehalose due to deletion of the trehalose-6-phosphate synthase gene showed that trehalose is neither necessary nor sufficient for survival of desiccation [70]. Therefore, the up-accumulation of trehalose in the relatively desiccation-sensitive *E. mirabilis*—and, to some extent, also in the aquatic *C. vulgaris* strain—compared to *D. epiphytica* and the terrestrial *C. vulgaris* strain—could be related to the regulation of sugar metabolism and argues against a pivotal role of trehalose in desiccation tolerance.

Furthermore, several amino acids, including the branched-chain amino acids valine, leucine, and isoleucine, were up-accumulated in *E. mirabilis* compared to the other three strains (Figure 5). Branched-chain amino acids contribute to target of rapamycin (TOR) activation and signalling, an evolutionarily conserved hub of nutrient sensing that impacts on protein synthesis [71]. Allantoin, a nitrogen-rich heterocyclic metabolite involved in purine metabolism, was also up-accumulated. Allantoin has a housekeeping role in nitrogen recycling and plant stress response [72,73], and in green algae, including *Chlorella* sp., allantoin can serve as a sole nitrogen source for growth [74]. Therefore, the seven up-accumulated amino acids and allantoin (Figure 5) could reflect a differential regulation of nutrient sensing and nitrogen assimilation in *E. mirabilis*, but more research is needed to elucidate the ecophysiological significance of this trait for *E. mirabilis*.

*Edaphochlorella mirabilis* and *D. epiphytica* had higher campesterol and stigmasterol contents but lower ergosterol contents as compared to the *C. vulgaris* strains (Figure 5). In higher plants, campesterol is a precursor of brassinolide, which promotes plant growth and also plays a role in stress resistance through interacting with abscisic acid signalling [75]. In microalgae, brassinosteroids have also been associated with responses to various abiotic stress factors, such as heavy metals, salt, and thermal stress [76]. Moreover, it has been noted a long time ago that sterols of green algae are much more varied and complex than those of other algae, and could be used as chemotaxonomic markers [77]. Interestingly, unicellular algae have recently been identified as an evolutionary transition point for sterols [78]. Sterols are found in distinctly different compositions throughout several kingdoms of life, with cholesterol being the main sterol in animals, ergosterol in fungi, and “phytosterols” such as stigmasterol, campesterol, and β-sitosterol being the dominant sterols in plant membranes. Sterols confer membrane stability, essentially contributing to the integrity, fluidity, and permeability of the lipid bilayer, and also affecting membrane-bound protein composition and influencing the functionality of enzymes, receptors, and channels [79]. An investigation into the distribution of ergosterol in 20 *Chlorella* species, some of which were later re-assigned to the *Prasiola* clade [6,7], showed that ergosterol was present in nine strains of Chlorellaceae only [80]. Furthermore, Chlorellaceae do not contain prasiolin (Table S2), a mycosporine-like amino acid related to UV protection, named after *Prasiola* sp. [48]. Although long assigned to the *Chlorella* genus, *D. epiphytica* and *E. mirabilis* belong to the *Prasiola* clade [6]. Hence, the differential accumulation of campesterol, ergosterol, and stigmasterol together with prasiolin likely reflects phylogeny rather than differences in stress response, with constitutively different membrane compositions in the two species in the *Prasiola* clade, *D. epiphytica*, and *E. mirabilis*, compared to the two *C. vulgaris* strains.

Aigner et al. [4] previously reported that the two *C. vulgaris* strains differ in their allocation of carbon and nitrogen into their primary metabolites, and in amino acid and polyamine metabolism. The meta-analysis of these two *C. vulgaris* strains together with *D. epiphytica* and *E. mirabilis* revealed that the aquatic *C. vulgaris* strain had an overall lower amino acids content as compared to the other three strains, with the lowest asparagine, lysine, methionine, threonine, glutamic acid, phenylalanine, glycine, and arginine contents. Proteinogenic amino acids are essential to synthesize chaperone-like proteins, which are important players in desiccation tolerance, as demonstrated for desiccation tolerant cyanobacteria [81]. In higher plants, arginine is required for polyamine synthesis, whereas unicellular green algae appear to depend on putrescine biosynthesis from ornithine, an arginine derivative [82]. The polyamine putrescine, often associated with plant stress tolerance [83,84], was also present at high levels in the terrestrial *C. vulgaris* strain. Putrescine is another compatible solute associated with mechanisms that protect from desiccation and freezing *via* stabilizing macromolecules and scavenging of reactive oxygen species [83], including in the photosynthetic apparatus [84]. The up-accumulation of glycine and serine together with increased levels of glycerate and glycolate in the terrestrial *C. vulgaris* strain was interpreted as an indication for a higher flux through the photorespiration pathway [4]. Unexpectedly, the aquatic *C. vulgaris* strain contained higher levels of the nonproteinogenic amino acid pipecolate, associated with osmoprotection, but also with disorders of lysine metabolism [85], compared to the three other strains. Recently, the acquisition of desiccation tolerance has been studied in *Haematococcus pluvialis*, which requires photosynthesis and coincides with lipid and astaxanthin accumulation [86]. However, the up-accumulation of myristic acid in the terrestrial *C. vulgaris* and linoleic acid in *D. epiphytica* and *C. vulgaris* should not be over-interpreted, as only free fatty acids were detected with GC–MS-based metabolite profiling. In summary, the metabolite profiles of the four algal strains reflect their phylogenic relationships, lifestyle (aquatic, terrestrial, and symbiotic), and tolerance of osmotic and/or desiccation stress.

### 4.2. Differences in Metabolite Profiles among the Four Algal Strains after Desiccation and Rehydration

Changes in metabolites associated with desiccation and rehydration have been shown for other desiccation-tolerant organisms (for review of detailed omics-analyses in resurrection plants, see [87]). For example, a comparison between a desiccation-tolerant and a desiccation-sensitive grass, *Sporobolus stapfianus* and *S. pyramidalis*, showed that *S. stapfianus* is metabolically primed for desiccation by up-accumulating osmolytes and nitrogen source compounds, and down-accumulating compounds related to energy metabolism and growth, compared to *S. pyramidalis* [88]. Furthermore, the desiccation-tolerant lycopod *Selaginella lepidophylla* retained higher amounts of sucrose, mono- and polysaccharides, and sugar alcohols such as sorbitol and xylitol than the desiccation-sensitive *Selaginella moellendorffii* [89]. Our present study contributes further knowledge of metabolite groups potentially involved in desiccation tolerance. Importantly, we show that the most desiccation-tolerant alga, *D. epiphytica*, had the best capability of either maintaining or restoring its metabolic state upon rehydration, whereas the most desiccation-sensitive alga, the aquatic *C. vulgaris* strain, appeared to be out of equilibrium, assessed by its greater variation in metabolite accumulation after the desiccation-rehydration treatment (Figure 6). Therefore, it appears that differences in relative desiccation tolerance are reflected in the metabolic recovery after desiccation and rehydration, and specifically, by the magnitude of differential metabolite accumulation.

Of the individual differentially accumulated metabolites, sucrose was unaffected in *E. mirabilis* and the terrestrial *C. vulgaris* strain, up-accumulated in *D. epiphytica*, and down-accumulated in the aquatic *C. vulgaris* (Figure 7), suggestive of stronger imbalances in energy metabolism in the latter strain, in line with poor recovery of photosynthesis [4], and could also point at a role of sucrose in desiccation tolerance. The aquatic *C. vulgaris* strain also up-accumulated compatible solutes such as putrescine and xylitol, and trehalose-6-phosphate, which could reflect that it had received a signal to up-regulate mechanisms that protect from desiccation-induced injury. It will be interesting to conduct future experiments to see if repeated desiccation-rehydration cycles can induce hardening in the aquatic *C. vulgaris* strain. Furthermore, the two strains with intermediate desiccation tolerance, the terrestrial *C. vulgaris* strain and *E. mirabilis*, showed very similar patterns of differential accumulation, with few exceptions, including maltose. This disaccharide was down-accumulated in *E. mirabilis* and up-accumulated in the terrestrial *C. vulgaris* strain after treatment, indicative of starch degradation, similar to that reported for algal cells in the top layer of a *Zygnema* mat exposed to desiccation and irradiation, in which an increase in maltose was shown by metabolite profiling and starch degradation by transmission electron microscopy [90]. However, assessing the precise roles of individual metabolites in desiccation tolerance is not straightforward. For example, putrescine was up-accumulated in *E. mirabilis* and the aquatic *C. vulgaris* strain and down-accumulated in the terrestrial *C. vulgaris* strain after desiccation and rehydration. Opposite accumulation patterns were also reported for some metabolites in the above-mentioned desiccation-tolerant and the desiccation-sensitive pairs of *Sporobolus* and *Selaginella* such as asparagine, aspartate, arginine, and glutamate [88,89].

## 5. Summary and Conclusions

The metabolite composition of the four algal strains investigated prior to the desiccation-rehydration experiment partly reflects their phylogenetic position, for example, sterol composition and prasiolin, whereas in *D. epiphytica*, the abundance of other metabolites also reflects its suitability for symbiosis with a lichenized fungus [58], especially sugars and sugar alcohols such as ribitol, erythritol, and sorbitol [31,32]. Furthermore, sugar alcohols also play important roles as compatible solutes protecting cellular structures from osmotic and desiccation stress [24,30,31,32], but it is not possible to associate stress tolerance to individual compatible solutes, as the four strains appear to use different metabolites for the same or similar purposes. A key finding of this study is that the smallest number of metabolites was significantly differentially accumulated in the most desiccation-tolerant strain, *D. epiphytica*, and the largest number in the least desiccation-tolerant strain, the aquatic *C. vulgaris*. This suggests that *D. epiphytica* had the best capability of either maintaining its primary metabolite composition required for metabolism prior to the treatment and/or re-adjusting its metabolism upon rehydration, in agreement with the full recovery of YII values of nondesiccated controls. By contrast, the metabolism of the aquatic *C. vulgaris* strain appeared to be more out of equilibrium, in agreement with the poor recovery of YII values of nondesiccated controls. Interestingly, the differential metabolite accumulation after the desiccation-rehydration experiment of the terrestrial *C. vulgaris* strain and *E. mirabilis* was in the middle of the two extremes, irrespective of their phylogenetic position in the *Chlorellaceae* and the *Prasiola* clade, respectively. Therefore, it appears that, at least for the four algal strains investigated here, the magnitude of differential metabolite accumulation after the desiccation-rehydration treatment reflects their degree of relative desiccation tolerance.

## Data Availability

Not applicable.

## Supplementary Materials

The following supporting information can be downloaded at: https://www.mdpi.com/article/10.3390/microorganisms10050946/s1, Table S1: Characteristic parameters of ETR curves of *Edaphochlorella mirabilis* and *Diplosphaera epiphytica*. Table S2: Concentrations of proline, sorbitol, and prasiolin in *Diplosphaera epiphytica* and *Edaphochlorella mirabilis*. Table S3: Complete dataset of metabolites measured by GC–MS.
