# Supplementary material for: Metabolite Profiling in Green Microalgae with Varying Degrees of Desiccation Tolerance
Source: Microorganisms. 2022 Apr 30;10(5):946. doi: 10.3390/microorganisms10050946 (PMC9144557; doi:10.3390/microorganisms10050946)
Supplement: Supplementary file 1 [file microorganisms-10-00946-s001.zip › Aigner_et_al_Table S1.pdf]

Table S1. Characteristic parameters of ETR curves of *Edaphochlorella mirabilis* and *Diplosphaera epiphytica*.

Species / treatment	ETR_{max}	I_k	α	β
D. epiphytica untreated	35.7 ± 5.5^B	125.2 ± 19.7^A	0.297 ± 0.008^A	-0.002 ± 0.002^A
D. epiphytica D/RH	23.3 ± 0.4^A	81.2 ± 3.8^B	0.304 ± 0.007^A	-0.003 ± 0.001^A
E. mirabilis untreated	25.7 ± 3.2^A	143.3 ± 11.0^A	0.179 ± 0.013^B	-
E. mirabilis D/RH	20.9 ± 2.4^A	135.1 ± 12.3^A	0.196 ± 0.030^B	-0.010 ± 0.001^B

D/RH denotes values after the desiccation-rehydration treatment. Data were calculated by using the fitting model according to Webb et al. 1974 or Walsby 1997 depending on either photoinhibition occurred or not (n = 4, mean value ± SD). ETR_{max}, maximum electron transport rate (μmol electrons m^{-2} s^{-1}), α initial slope at limiting photofluence rates (electrons $photon^{-1}$), I_k initial value of light-saturated photosynthesis (μmol photons m^{-2} s^{-1}). Different capital letters indicate significant differences and were calculated by two-way ANOVA followed by Tukey's post hoc test ($P < 0.05$).
