# Supplementary material for: Metabolite Profiling in Green Microalgae with Varying Degrees of Desiccation Tolerance
Source: Microorganisms. 2022 Apr 30;10(5):946. doi: 10.3390/microorganisms10050946 (PMC9144557; doi:10.3390/microorganisms10050946)
Supplement: Supplementary file 1 [file microorganisms-10-00946-s001.zip › Aigner_et_al_Table S2.pdf]

Table S2. Concentrations of proline, sorbitol and prasiolin in *Edaphochlorella mirabilis* and *Diplosphaera epiphytica* (n = 3, mean value ± SD). Proline was measured spectrophotometrically and sorbitol and prasiolin by HPLC; concentrations are given in µmol g^{-1} dry weight. Proline and prasiolin concentrations were significantly different between the two strains ($P < 0.05$).

Metabolite	*E. mirabilis*	*D. epiphytica*
Proline	64.5 ± 8.91	152.6 ± 9.05
Sorbitol	n.d.	168.2 ± 21.5
Prasiolin	3.6 ± 0.06	17.2 ± 0.96

n.d.: not detected.
